# Effects of Vonoprazan Compared with Esomeprazole on the Healing of Artificial Postendoscopic Submucosal Dissection Ulcers: A Prospective, Multicenter, Two-Arm, Randomized Controlled Trial

**DOI:** 10.1155/2018/1615092

**Published:** 2018-02-18

**Authors:** Yasuaki Ishii, Hiroaki Yamada, Takeshi Sato, Soichiro Sue, Hiroaki Kaneko, Kuniyasu Irie, Tomohiko Sasaki, Toshihide Tamura, Ryosuke Ikeda, Takehide Fukuchi, Ryosuke Kobayashi, Makomo Makazu, Chiko Sato, Kingo Hirasawa, Masaaki Kondo, Wataru Shibata, Shin Maeda

**Affiliations:** ^1^Department of Gastroenterology, Yokohama City University Graduate School of Medicine, 3-9 Fukuura, Kanazawa-ku, Yokohama 236-0004, Japan; ^2^Division of Endoscopy, Yokohama City University Medical Center, 4-57 Urafune-cho, Minami-ku, Yokohama 232-0024, Japan; ^3^Advanced Medical Research Center, Yokohama City University, 3-9 Fukuura, Kanazawa-ku, Yokohama 236-0004, Japan

## Abstract

**Background:**

Vonoprazan affords more clinical benefits than proton pump inhibitors (PPIs) during the healing of gastroduodenal ulcers. However, it remains controversial whether vonoprazan is more effective than PPIs when used to heal artificial ulcers arising after endoscopic submucosal dissection (ESD).

**Aim:**

This study investigated the effects of vonoprazan compared with esomeprazole on the healing of post-ESD artificial ulcers.

**Methods:**

Sixty patients who underwent gastric ESD between May 2015 and May 2017 were randomized to treatment with vonoprazan (V group) or esomeprazole (E group) for 8 weeks. Upper endoscopy was performed at 4 and 8 weeks after ESD, and drug effects were estimated based on the ulcer healing rates and shrinkage rates.

**Results:**

Fifty-three patients were analyzed. The respective 4- and 8-week ulcer healing rates did not differ significantly between V and E groups (8.0 versus 11.5%, *P* = 0.669; 88.9 versus 84.6%, *P* = 0.420). Similarly, the respective 4- and 8-week ulcer shrinkage rates did not differ significantly between V and E groups (96.8 versus 97.5%, *P* = 0.656; 100 versus 100%, *P* = 0.257).

**Conclusion:**

The healing of artificial ulcers after ESD did not differ using vonoprazan or esomeprazole. Both vonoprazan and esomeprazole were effective when used to promote artificial ulcer healing after ESD.

## 1. Introduction

Endoscopic submucosal dissection (ESD), which was developed in Japan in the late 1990s, has been performed in many countries in recent years; with ESD, the 5-year survival rate of patients with early gastric cancer (EGC) exceeds 90% [1].

Several complications of ESD are known, the most important of which is post-ESD bleeding [2]. As ulcer healing prevents post-ESD bleeding, proton pump inhibitors (PPIs) are widely prescribed as the first-line therapy for artificial ulcers developing after ESD [3–5].

Recently, a novel potassium-competitive acid blocker (P-CAB) termed vonoprazan (TAKECAB; Takeda Pharmaceutical Co. Ltd., Tokyo, Japan) was developed. P-CAB exhibits a more powerful and longer antisecretory effect on H^+^/K^+^-ATPase than do PPIs. P-CAB was reported to be more effective than PPIs in the healing of gastroduodenal ulcers [6, 7]. Thus, P-CAB would be expected to afford better healing of artificial ulcers developing after ESD.

We began to investigate the effect of P-CAB on the healing of post-ESD artificial ulcers in March 2015 (trial UMIN000016835). To date, four comparative studies have examined the extent of artificial ulcer healing afforded by P-CAB compared with PPIs [8–11]. However, the results were controversial, and further work was required. Here, we examined the effects of vonoprazan compared with those of esomeprazole on the healing of post-ESD artificial ulcers in a prospective, multicenter, two-arm, randomized controlled trial (RCT) and found that the extent of healing of artificial ulcers after ESD was identical when either P-CAB or PPI was prescribed.

## 2. Methods

### 2.1. Study Design and Patients

We conducted a prospective study between May 2015 and May 2017 at two university hospitals (Yokohama City University Hospital, Yokohama, Japan, and Yokohama City University Medical Center, Yokohama, Japan). The study protocol was approved by the ethics review boards of both hospitals, and the trial was registered with the University Hospital Medical Information Network (number MIN000016835) and performed in accordance with the Declaration of Helsinki.

Patients diagnosed with EGC or gastric adenoma and treated via ESD at either hospital were recruited. We included patients who were ≥ 20 years of age and provided written informed consent. Conversely, our exclusion criteria were (i) continuous prescription of any medicine that could interact with vonoprazan or esomeprazole (e.g., another PPI or an H2 receptor blocker); (ii) prescription of NSAIDs, steroids, anticoagulants, and/or antithrombotic agents; (iii) pregnancy; (iv) any serious disease rendering ESD difficult; (v) a past history of resection of the upper gastrointestinal tract; or (vi) considered incompetent by a doctor.

**Figure 1:**
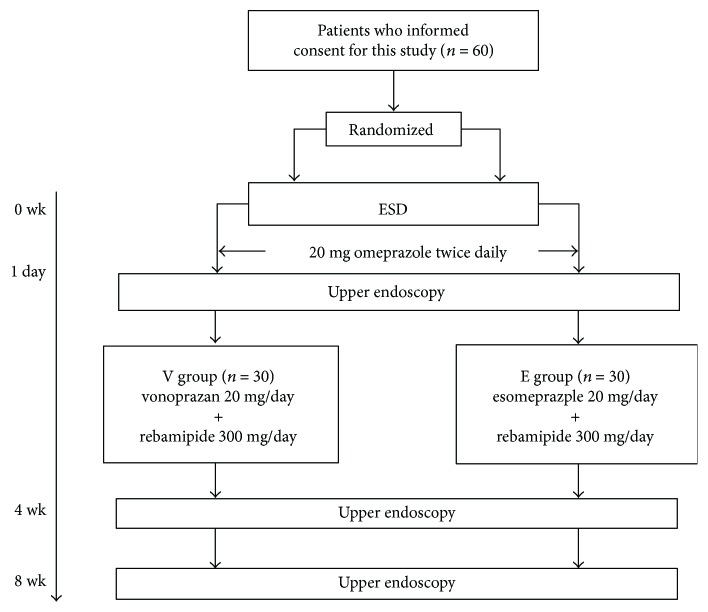
Patient flow chart. Sixty patients who gave written informed consent were randomly divided into two groups. All patients were given injections of 20 mg of omeprazole on the day of ESD and the next day. Oral administration of the test medications commenced on day 2 after ESD. Ulcer areas were evaluated via upper endoscopy on the next day and 4 weeks and 8 weeks after ESD, respectively.

**Figure 2:**
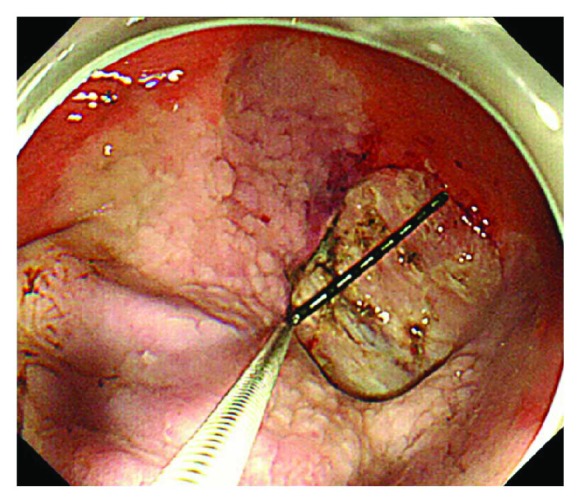
The major and minor axes of the artificial ulcers were measured endoscopically.

A total of 60 patients were randomly (and equally) divided into a vonoprazan group (V group) and an esomeprazole group (E group) using QMinim Online Minimization (http://qminim.sourceforge.net) prior to ESD. We divided age, sex, *Helicobacter pylori* infection, and diabetes into stratification. Neither the physicians nor the patients were blinded to group status. All patients were given injections of 20 mg of omeprazole twice daily on the day of ESD and on the next day. Two days after ESD, 20 mg of vonoprazan and 300 mg of rebamipide (V group) or 20 mg of esomeprazole and 300 mg of rebamipide (E group) were prescribed orally (daily) for 8 weeks. E group is the regular follow-up of each hospital. To evaluate the sizes and conditions of all artificial ulcers, the patients underwent upper endoscopy on the day after ESD and at 4 and 8 weeks later (Figure 1). The major and minor axes of the ulcers were measured endoscopically (M2-4K; Olympus Corp., Tokyo, Japan) (Figure 2). Assuming that each ulcer was an ellipse, the ulcer area (in mm^2^) was calculated using the following formula [12]: (major axis/2) × (minor axis/2) × *π*.

### 2.2. ESD

We performed ESD using a single-channel endoscope (GIF-Q260J; Olympus Corp.) or via multiangle two-channel endoscopy (GIF-2TQ260M; Olympus Corp.). The injection solution contained glycerol, hyaluronic acid sodium (0.4% [*w*/*v*]), and 0.001% (*w*/*v*) epinephrine and was locally injected into the submucosal layer using a disposable 23-gauge needle (Top Corp., Tokyo, Japan). The ITknife2 (KD-611L; Olympus Corp.) was the primary cutting device used, but we occasionally employed a DualKnife (KD-650U; Olympus Corp.). An electrosurgical current was applied with the aid of an electrosurgical generator (VIO300D or ICC200; ERBE Elektromedizin GmbH, Tubingen, Germany). Ulcers that developed after ESD were carefully examined endoscopically and any visible vessels clipped (EZ Clip; Olympus Corp.) and/or heat-coagulated using hemostatic forceps (Coagrasper, FD410LR; Olympus Corp.) in all patients in both groups.

### 2.3. Endpoint

Our primary endpoint was the shrinkage rate of the artificial ulcers 4 and 8 weeks after ESD. The shrinkage rate was calculated using the following formula: ([ESD specimen area] − [ulcerated area at 4 or 8 weeks after ESD])/(ESD specimen area) × 100(%). The ulcer healing rate (scarring at stage S1 or S2) was also noted. In addition, we compared the post-ESD bleeding rates. Post-ESD bleeding was defined as a clinical episode of hematemesis and/or melena and/or a decline in the hemoglobin level to below 2 g/dL.

### 2.4. Statistical Analysis

We used Fisher's exact test to compare quantitative variables (sex, *Helicobacter pylori* infection status, diabetes, and tumor location). The Mann–Whitney *U* test was employed to compare age, ulcer area, and ulcer cure rate. *P* values < 0.05 were considered to reflect statistical significance. Patient ages and ESD specimen areas are presented as medians with interquartile ranges (IQRs). The IQR is a measure of statistical dispersion, being the difference between the 75th and 25th percentiles, or between the upper and lower quartiles.

All statistical analyses were performed with the aid of EZR software (Saitama Medical Center, Jichi Medical University, Saitama, Japan), which is a graphical user interface for R (The R Foundation for Statistical Computing, Vienna, Austria). More precisely, EZR is a modified version of the R commander with additional statistical functions used frequently by biostatisticians [13].

## 3. Results

### 3.1. Characteristics of the Patients and Lesions

In consideration of the cure healing rate of vonoprazan and esomeprazole in the previous report, it was judged that there was a significant difference in 72 patients, and this study was conducted. Intermediate analysis was carried out when 60 patients finished, because there was no difference, this study was terminated. Their distributions between the two participating institutions are shown in Table 1. We randomly divided the enrolled patients into two groups of 30 patients each. We excluded 3 patients of the V group and 4 patients of the E group, finally analyzing 27 and 26 patients, respectively. The reasons for exclusion in the V group (*n* = 3) were surgical treatment (*n* = 1), a suspected allergic reaction (*n* = 1), and a perforation (*n* = 1); in the E group (*n* = 4), the reasons for exclusion were surgical treatment (*n* = 3) and loss to follow-up as defined in the protocol (*n* = 1). Drug compliance exceeded 90% in all patients. Several clinical characteristics that delay ulcer healing have been reported [5, 14–16]. Therefore, we analyzed the data with respect to age, sex, *H. pylori* infection status, the presence of diabetes mellitus, and tumor location but found no significant difference between the two groups in terms of any factor (Table 2). We also analyzed tumor size, specimen size, and procedure duration, which are known as risk factors of post-ESD bleeding [2], but found no significant difference between the two groups in terms of any factor (Table 2). *H. pylori* infection rate in this study was lower than that of Japanese general population, because *H. pylori* has been eradicated before ESD in some patients.

### 3.2. Ulcer Healing and Shrinkage Rate

**Figure 3:**
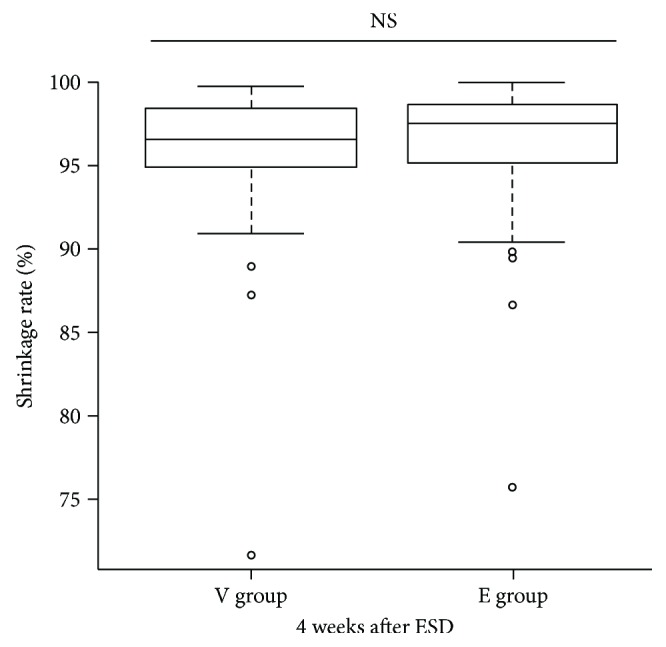
The shrinkage rates of ulcers of the V group and E group 4 weeks after ESD.

The median areas of the ESD artificial ulcers were 961.8 (IQR: 707.7–1380.0) mm^2^ and 880.8 (IQR: 588.8–1638.8) mm^2^ in the V group and E group, respectively. We performed endoscopic follow-up at 4 and 8 weeks after ESD. The proportions of subjects with ulcer scarring of stage S1 or S2 after ESD were 8.0% (2/27) and 11.5% (3/26) at 4 weeks (*P* = 0.669) and 88.9% (24/27) and 84.6% (22/26) at 8 weeks (*P* = 0.420) in the V and E groups, respectively (Table 3). The 4-week shrinkage rates of artificial ulcers were 96.8% (range: 72.0–100%) in the V group and 97.5% (range: 75.8–100%) in the E group, thus not significantly different between the V and E groups (*P* = 0.656) (Figure 3). The 8-week shrinkage rates of artificial ulcers were 100% in both groups, thus lacking any significant difference (*P* = 0.257) (Table 3). In terms of delayed bleeding, no obvious bleeding was observed in either group. As the healing of artificial ulcers after ESD did not differ when vonoprazan or esomeprazole was prescribed, both drugs effectively aided artificial ulcer healing after ESD.

## 4. Discussion

We sought to clarify the effects of vonoprazan and esomeprazole on the healing of post-ESD artificial ulcers by designing a prospective, multicenter, two-arm RCT. We found no significant difference between vonoprazan and esomeprazole in terms of artificial ulcer healing after ESD. Statistically, no factor associated with delayed ulcer healing was evident. As the study was performed at two centers, nine physicians (ranging from beginners to experts) performed ESD, but their various skill levels did not affect the results.

Of the four previous reports comparing artificial ulcer healing using P-CAB and PPIs, three found that P-CAB was superior to PPIs in terms of post-ESD ulcer shrinkage rates [10, 11] or late bleeding rates [8]. However, no obvious differences in ulcer shrinkage rate were found in either our present study or another report [9]. Differences among the results of the five studies (including this study) are probably due to differences in protocols, patient selection, and other variables. We excluded patients who were taking NSAIDs, steroids, anticoagulants, and/or antithrombotic agents to minimize situations where other medications might influence the effects of P-CAB or PPIs. Therefore, it may be that we found no significant difference because we selectively excluded patients with comorbidities (which the other studies did not). In particular, excluding patients on antithrombotic therapy may explain the absence of bleeding in our study [17]. Although we cannot mention about post-ESD bleeding assertively in our study, we analyzed several factors (tumor size, specimen size, and procedure duration), which are known as risk factors of post-ESD bleeding [2]. As a result, there was no significant difference in any of them (Table 2). Another difference is that our study did not feature a monotherapy protocol; we added oral rebamipide because a previous meta-analysis showed that treatment with PPIs plus rebamipide was superior to PPIs monotherapy in terms of the healing of ESD-induced ulcers over 4 weeks, particularly large ulcers [18]. As patients should not be disadvantaged during clinical trials, we considered that monotherapy would not be ethical. Also, the median ESD specimen area in the present study was only 942 mm^2^, thus small compared to those of previous reports (1256 mm^2^ [10] and 1114.7 mm^2^ [11]). The low rate of *H. pylori* infection may also be a factor that did not differ significantly in ulcer shrinkage rate.

Vonoprazan action is barely affected by the CYP2C19 genotype, and the drug affords more potent and longer antisecretory effects on H^+^/K^+^-ATPase than do conventional PPIs [19, 20]. Therefore, vonoprazan has been widely prescribed in Japan for the treatment of gastric ulcers, for *H. pylori* eradication, and for the treatment of gastroesophageal reflux disease in the time since its release in 2015. Although vonoprazan inhibits acid production irrespective of CYP2C19 status more potently than does esomeprazole [20], CYP2C19 status is associated with approximately a threefold lower intrinsic clearance of the S-isomer of esomeprazole compared with the R-isomer and omeprazole. Esomeprazole is less affected by metabolism than are the other PPIs [21]. In fact, as we found no significant difference between the drugs in the present study, it may be unnecessary to strongly inhibit gastric acid secretion during artificial ulcer healing.

The limitations of our study include the mode of administration of drugs and the method that we used to measure ulcer area. We did not simply compare the effects of vonoprazan and esomeprazole because we gave rebamipide and omeprazole intravenously. However, we considered that we should not disadvantage the patients. Also, monotherapy is not prescribed in actual clinical practice. As various treatments are available, we concluded that our protocol was the most appropriate. We earlier thoroughly evaluated the method that we used to measure ulcers. The stomach can be easily expanded and contracted by supplying, and then removing, gas via an endoscope. However, the stomach is of course a three-dimensional structure. As endoscopic images can be captured from flat surfaces only, the accuracies of our area measurements may have been compromised. The reason that we used our previously reported method [12] is that the region just after the ESD lies closest to the ESD sample area (data not shown).

In conclusion, we found no difference in terms of artificial ulcer healing after ESD using rebamipide in combination with either vonoprazan or esomeprazole in low-risk patients with late bleeding. Vonoprazan strongly suppresses gastric acid secretion, but there was no great need for the use of this drug during the healing of artificial ulcers after ESD; esomeprazole exerted a sufficient therapeutic effect. No post-ESD bleeding was observed. We consider that both vonoprazan and esomeprazole effectively aided artificial ulcer healing and esomeprazole was superior to vonoprazan in view of cost benefit in low-risk patients.

## Figures and Tables

**Table 1:** Participating institutions and their contributions to the two patient groups.

Participating institutions	V group (exclude)	E group (exclude)
Yokohama City University Hospital	7 (1)	11 (4)
Yokohama City University Medical Center	23 (2)	19 (0)
Total	30 (3)	30 (4)

**Table 2:** Characteristics of all parameters analyzed.

Characteristic	Total (*n* = 53)	V group (*n* = 27)	E group (*n* = 26)	*P* value
Age (years) median (IQR)	70.2 (66.76)	70 (65.3–75)	70 (66–75.3)	0.957^∗∗^
Sex (male), *n* (%)	55 (87.3)	23 (85.2)	22 (84.6)	0.728^∗^
*Helicobacter pylori* infection (positive), *n* (%)	15 (28.3)	7 (26.0)	8 (30.8)	0.766^∗^
Diabetes, *n* (%)	8 (12.7)	3 (11.1)	5 (19.2)	0.467^∗^
Location (upper, middle, lower)	28, 20, 7	12, 10, 5	14, 10, 2	0.596^∗^
Location (greater curvature, lesser curvature, anterior wall, posterior wall)	13, 7, 11, 10	7, 13, 3, 4	6, 6, 8, 6	0.157^∗^
Tumor size (mm), range, *n* (>20 mm)	14.1 (4–38), 12	14.9 (4–38), 7	13.2 (4–35), 5	0.745^∗^
Specimen size (mm), range, *n* (>30 mm)	40.2 (26–80), 40	40.6 (30–54), 22	39.8 (26–80), 18	0.352^∗^
Procedure duration (min), range, *n* (>60 min)	43.3 (8–103), 14	45.5 (9–103), 8	41.1 (8–100), 6	0.757^∗^

IQR: interquartile range (a measure of variability, based on dividing a dataset into quartiles). A *P* value < 0.05 was considered to reflect significance. ^∗^Significant by Fisher's exact test. ^∗∗^Significant by the Mann–Whitney *U* test.

**Table 3:** Ulcer evaluations after ESD, and the absence of post-ESD bleeding.

	Total (*n* = 53)	V group (*n* = 27)	E group (*n* = 26)	*P* value
Ulcer stage after 4 weeks (scar, *n*)	5	2	3	0.669^∗∗^
Ulcer stage after 8 weeks (scar, *n*)	46	24	22	0.42^∗∗^
Shrinkage rate after 4 weeks (%), range	95.4 (72.0–100)	96.8 (72.0–100)	97.5 (75.8–100)	0.656^∗∗^
Shrinkage rate after 8 weeks (%)	100	100	100	0.257^∗∗^
ESD specimen area (mm^2^) median (IQR)	942.3 (588.8–1452.3)	961.8 (707.8–1380.0)	880.8 (588.8–1638.8)	0.612^∗∗^
Post-ESD bleeding	0	0	0	

IQR: interquartile range (a measure of variability, based on dividing a dataset into quartiles). A *P* value < 0.05 was considered significant. ^∗∗^Significant by the Mann–Whitney *U* test.
